# 

**Published:** 1971-01

**Authors:** 


Bristol Medico-Chirurgical Journal

Vol. 86 (i) No. 317

### Contents

Examinations using Multiple Choice Questions ... ... ... 3
R. St. J. Buxton

"There's Safety in Numbers" ... ... ... ... ... ... 7

Alan B. Raper

Obituary ... ... ... ... ... ... ... ... ... 11

Wisdom from the Past ... ... ... ... ... ... ... 13

Medical Library ... ... ... ... ... ... ... ... 15

Society Reports ... ... ... ... ... ... ... ... 17

F. Bailey & Son Ltd., Dursley, Glos.

